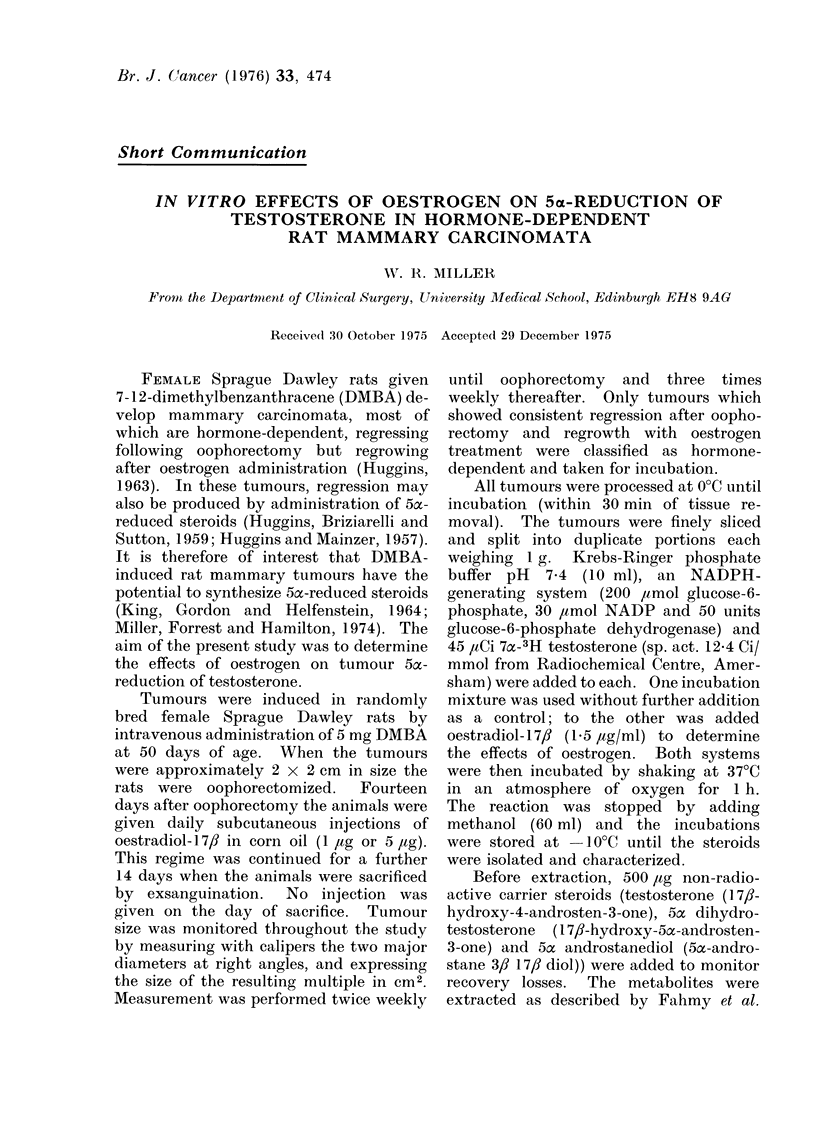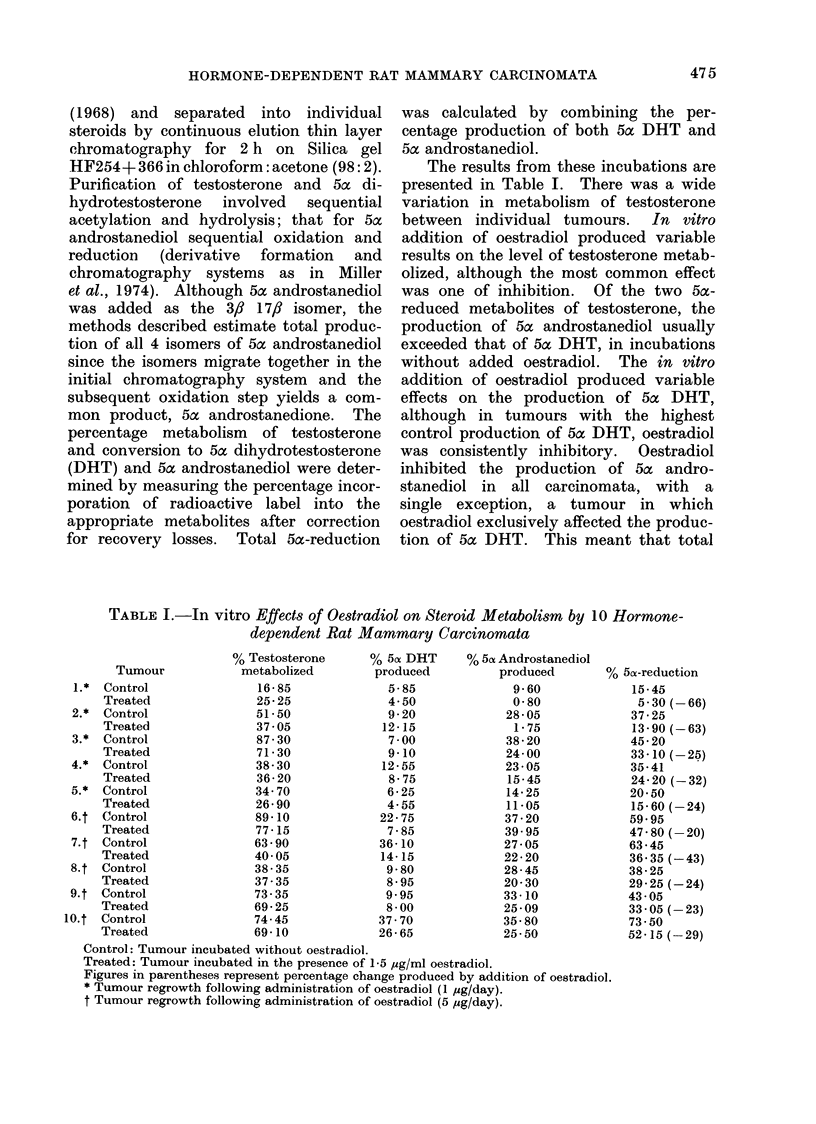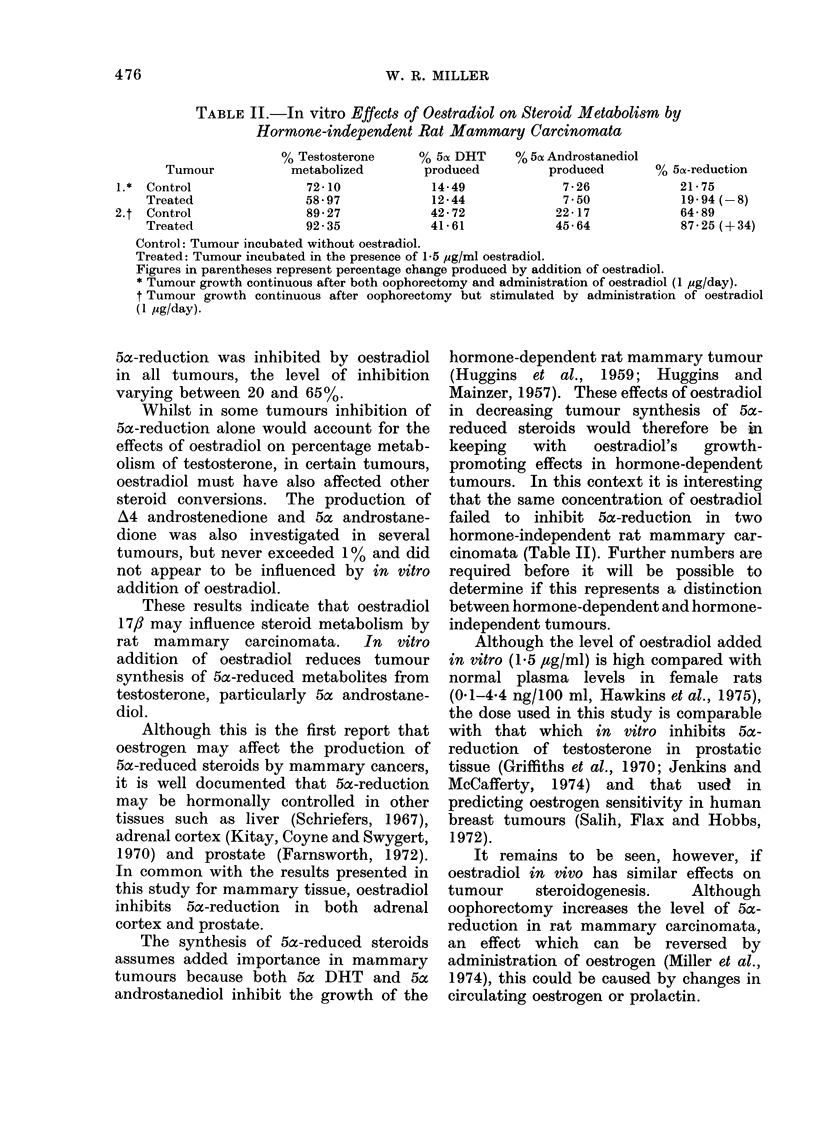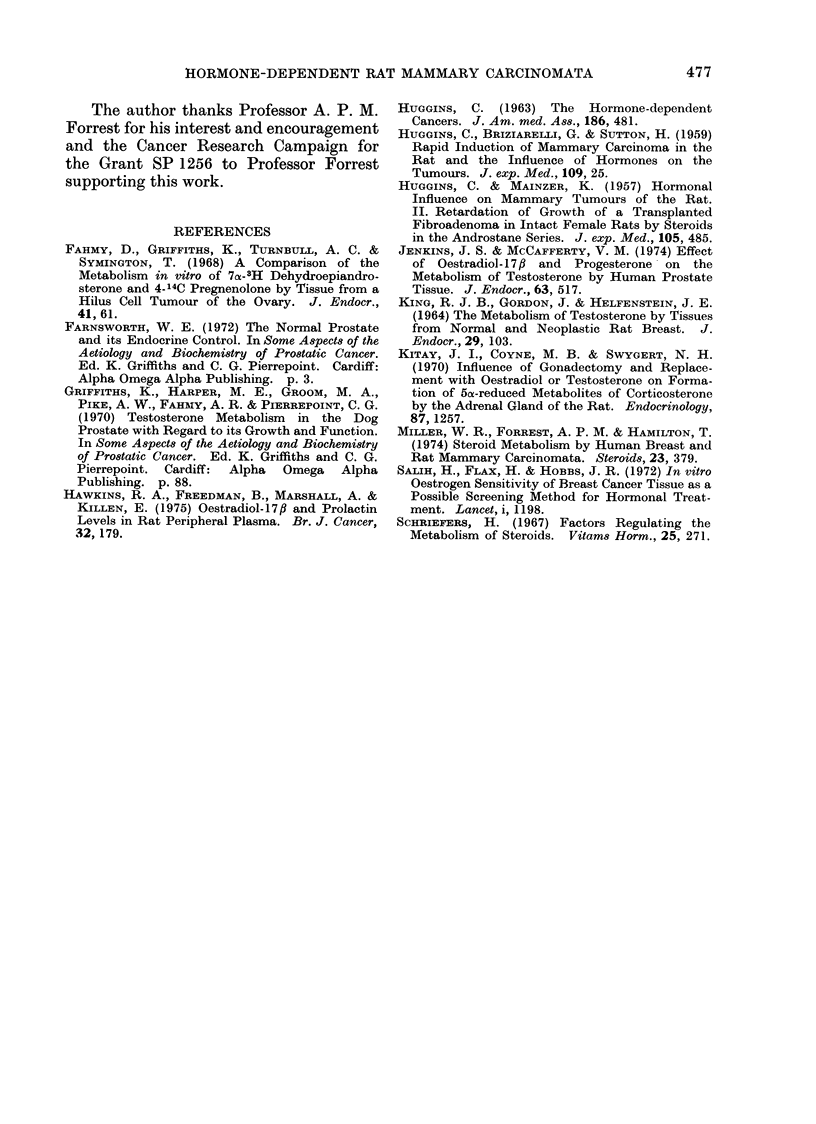# In vitro effects of oestrogen on 5alpha reduction of testosterone in hormone-dependent rat mammary carcinomata.

**DOI:** 10.1038/bjc.1976.74

**Published:** 1976-04

**Authors:** W. R. Miller


Br. J. Cancer (1976) 33, 474

*Short Communication*

### *IN VITRO* EFFECTS OF OESTROGEN ON 5α-REDUCTION OF TESTOSTERONE IN HORMONE-DEPENDENT RAT MAMMARY CARCINOMATA

W. R. MILLER

*From the Department of Clinical Surgery, University Medical School, Edinburgh EH8 9AG*

Received 30 October 1975 Accepted 29 December 1975

FEMALE Sprague Dawley rats given 7-12-dimethylbenzanthracene (DMBA) develop mammary carcinomata, most of which are hormone-dependent, regressing following oophorectomy but regrowing after oestrogen administration (Huggins, 1963). In these tumours, regression may also be produced by administration of 5α-reduced steroids (Huggins, Briziarelli and Sutton, 1959; Huggins and Mainzer, 1957). It is therefore of interest that DMBA-induced rat mammary tumours have the potential to synthesize 5α-reduced steroids (King, Gordon and Helfenstein, 1964; Miller, Forrest and Hamilton, 1974). The aim of the present study was to determine the effects of oestrogen on tumour 5α-reduction of testosterone.

Tumours were induced in randomly bred female Sprague Dawley rats by intravenous administration of 5 mg DMBA at 50 days of age. When the tumours were approximately 2 × 2 cm in size the rats were oophorectomized. Fourteen days after oophorectomy the animals were given daily subcutaneous injections of oestradiol-17β in corn oil (1 μg or 5 μg). This regime was continued for a further 14 days when the animals were sacrificed by exsanguination. No injection was given on the day of sacrifice. Tumour size was monitored throughout the study by measuring with calipers the two major diameters at right angles, and expressing the size of the resulting multiple in $cm^2$. Measurement was performed twice weekly until oophorectomy and three times weekly thereafter. Only tumours which showed consistent regression after oophorectomy and regrowth with oestrogen treatment were classified as hormone-dependent and taken for incubation.

All tumours were processed at 0°C until incubation (within 30 min of tissue removal). The tumours were finely sliced and split into duplicate portions each weighing 1 g. Krebs-Ringer phosphate buffer pH 7·4 (10 ml), an NADPH-generating system (200 μmol glucose-6-phosphate, 30 μmol NADP and 50 units glucose-6-phosphate dehydrogenase) and 45 μCi 7α-$^3$H testosterone (sp. act. 12·4 Ci/mmol from Radiochemical Centre, Amersham) were added to each. One incubation mixture was used without further addition as a control; to the other was added oestradiol-17β (1·5 μg/ml) to determine the effects of oestrogen. Both systems were then incubated by shaking at 37°C in an atmosphere of oxygen for 1 h. The reaction was stopped by adding methanol (60 ml) and the incubations were stored at −10°C until the steroids were isolated and characterized.

Before extraction, 500 μg non-radioactive carrier steroids (testosterone (17β-hydroxy-4-androsten-3-one), 5α dihydrotestosterone (17β-hydroxy-5α-androsten-3-one) and 5α androstanediol (5α-androstane 3β 17β diol)) were added to monitor recovery losses. The metabolites were extracted as described by Fahmy *et al.*

(1968) and separated into individual steroids by continuous elution thin layer chromatography for 2 h on Silica gel HF254+366 in chloroform : acetone (98 : 2). Purification of testosterone and 5α dihydrotestosterone involved sequential acetylation and hydrolysis; that for 5α androstanediol sequential oxidation and reduction (derivative formation and chromatography systems as in Miller *et al.*, 1974). Although 5α androstanediol was added as the 3β 17β isomer, the methods described estimate total production of all 4 isomers of 5α androstanediol since the isomers migrate together in the initial chromatography system and the subsequent oxidation step yields a common product, 5α androstanedione. The percentage metabolism of testosterone and conversion to 5α dihydrotestosterone (DHT) and 5α androstanediol were determined by measuring the percentage incorporation of radioactive label into the appropriate metabolites after correction for recovery losses. Total 5α-reduction was calculated by combining the percentage production of both 5α DHT and 5α androstanediol.

The results from these incubations are presented in Table I. There was a wide variation in metabolism of testosterone between individual tumours. *In vitro* addition of oestradiol produced variable results on the level of testosterone metabolized, although the most common effect was one of inhibition. Of the two 5α-reduced metabolites of testosterone, the production of 5α androstanediol usually exceeded that of 5α DHT, in incubations without added oestradiol. The *in vitro* addition of oestradiol produced variable effects on the production of 5α DHT, although in tumours with the highest control production of 5α DHT, oestradiol was consistently inhibitory. Oestradiol inhibited the production of 5α androstanediol in all carcinomata, with a single exception, a tumour in which oestradiol exclusively affected the production of 5α DHT. This meant that total

TABLE I.—In vitro *Effects of Oestradiol on Steroid Metabolism by* 10 *Hormone-dependent Rat Mammary Carcinomata*

| Tumour | | % Testosterone metabolized | % 5α DHT produced | % 5α Androstanediol produced | % 5α-reduction |
|---|---|---|---|---|---|
| 1.* | Control | 16·85 | 5·85 | 9·60 | 15·45 |
| | Treated | 25·25 | 4·50 | 0·80 | 5·30 (−66) |
| 2.* | Control | 51·50 | 9·20 | 28·05 | 37·25 |
| | Treated | 37·05 | 12·15 | 1·75 | 13·90 (−63) |
| 3.* | Control | 87·30 | 7·00 | 38·20 | 45·20 |
| | Treated | 71·30 | 9·10 | 24·00 | 33·10 (−25) |
| 4.* | Control | 38·30 | 12·55 | 23·05 | 35·41 |
| | Treated | 36·20 | 8·75 | 15·45 | 24·20 (−32) |
| 5.* | Control | 34·70 | 6·25 | 14·25 | 20·50 |
| | Treated | 26·90 | 4·55 | 11·05 | 15·60 (−24) |
| 6.† | Control | 89·10 | 22·75 | 37·20 | 59·95 |
| | Treated | 77·15 | 7·85 | 39·95 | 47·80 (−20) |
| 7.† | Control | 63·90 | 36·10 | 27·05 | 63·45 |
| | Treated | 40·05 | 14·15 | 22·20 | 36·35 (−43) |
| 8.† | Control | 38·35 | 9·80 | 28·45 | 38·25 |
| | Treated | 37·35 | 8·95 | 20·30 | 29·25 (−24) |
| 9.† | Control | 73·35 | 9·95 | 33·10 | 43·05 |
| | Treated | 69·25 | 8·00 | 25·09 | 33·05 (−23) |
| 10.† | Control | 74·45 | 37·70 | 35·80 | 73·50 |
| | Treated | 69·10 | 26·65 | 25·50 | 52·15 (−29) |

Control: Tumour incubated without oestradiol.
Treated: Tumour incubated in the presence of 1·5 μg/ml oestradiol.
Figures in parentheses represent percentage change produced by addition of oestradiol.
* Tumour regrowth following administration of oestradiol (1 μg/day).
† Tumour regrowth following administration of oestradiol (5 μg/day).

TABLE II.—In vitro *Effects of Oestradiol on Steroid Metabolism by Hormone-independent Rat Mammary Carcinomata*

| | Tumour | % Testosterone metabolized | % 5α DHT produced | % 5α Androstanediol produced | % 5α-reduction |
|---|---|---|---|---|---|
| 1.* | Control | 72·10 | 14·49 | 7·26 | 21·75 |
| | Treated | 58·97 | 12·44 | 7·50 | 19·94 (−8) |
| 2.† | Control | 89·27 | 42·72 | 22·17 | 64·89 |
| | Treated | 92·35 | 41·61 | 45·64 | 87·25 (+34) |

Control: Tumour incubated without oestradiol.
Treated: Tumour incubated in the presence of 1·5 μg/ml oestradiol.
Figures in parentheses represent percentage change produced by addition of oestradiol.
* Tumour growth continuous after both oophorectomy and administration of oestradiol (1 μg/day).
† Tumour growth continuous after oophorectomy but stimulated by administration of oestradiol (1 μg/day).

5α-reduction was inhibited by oestradiol in all tumours, the level of inhibition varying between 20 and 65%.

Whilst in some tumours inhibition of 5α-reduction alone would account for the effects of oestradiol on percentage metabolism of testosterone, in certain tumours, oestradiol must have also affected other steroid conversions. The production of Δ4 androstenedione and 5α androstanedione was also investigated in several tumours, but never exceeded 1% and did not appear to be influenced by *in vitro* addition of oestradiol.

These results indicate that oestradiol 17β may influence steroid metabolism by rat mammary carcinomata. *In vitro* addition of oestradiol reduces tumour synthesis of 5α-reduced metabolites from testosterone, particularly 5α androstanediol.

Although this is the first report that oestrogen may affect the production of 5α-reduced steroids by mammary cancers, it is well documented that 5α-reduction may be hormonally controlled in other tissues such as liver (Schriefers, 1967), adrenal cortex (Kitay, Coyne and Swygert, 1970) and prostate (Farnsworth, 1972). In common with the results presented in this study for mammary tissue, oestradiol inhibits 5α-reduction in both adrenal cortex and prostate.

The synthesis of 5α-reduced steroids assumes added importance in mammary tumours because both 5α DHT and 5α androstanediol inhibit the growth of the hormone-dependent rat mammary tumour (Huggins *et al.*, 1959; Huggins and Mainzer, 1957). These effects of oestradiol in decreasing tumour synthesis of 5α-reduced steroids would therefore be in keeping with oestradiol's growth-promoting effects in hormone-dependent tumours. In this context it is interesting that the same concentration of oestradiol failed to inhibit 5α-reduction in two hormone-independent rat mammary carcinomata (Table II). Further numbers are required before it will be possible to determine if this represents a distinction between hormone-dependent and hormone-independent tumours.

Although the level of oestradiol added *in vitro* (1·5 μg/ml) is high compared with normal plasma levels in female rats (0·1–4·4 ng/100 ml, Hawkins *et al.*, 1975), the dose used in this study is comparable with that which *in vitro* inhibits 5α-reduction of testosterone in prostatic tissue (Griffiths *et al.*, 1970; Jenkins and McCafferty, 1974) and that used in predicting oestrogen sensitivity in human breast tumours (Salih, Flax and Hobbs, 1972).

It remains to be seen, however, if oestradiol *in vivo* has similar effects on tumour steroidogenesis. Although oophorectomy increases the level of 5α-reduction in rat mammary carcinomata, an effect which can be reversed by administration of oestrogen (Miller *et al.*, 1974), this could be caused by changes in circulating oestrogen or prolactin.

The author thanks Professor A. P. M. Forrest for his interest and encouragement and the Cancer Research Campaign for the Grant SP 1256 to Professor Forrest supporting this work.

### REFERENCES

FAHMY, D., GRIFFITHS, K., TURNBULL, A. C. & SYMINGTON, T. (1968) A Comparison of the Metabolism *in vitro* of 7α-$^{3}$H Dehydroepiandrosterone and 4-$^{14}$C Pregnenolone by Tissue from a Hilus Cell Tumour of the Ovary. *J. Endocr.*, **41**, 61.

FARNSWORTH, W. E. (1972) The Normal Prostate and its Endocrine Control. In *Some Aspects of the Aetiology and Biochemistry of Prostatic Cancer.* Ed. K. Griffiths and C. G. Pierrepoint. Cardiff: Alpha Omega Alpha Publishing. p. 3.

GRIFFITHS, K., HARPER, M. E., GROOM, M. A., PIKE, A. W., FAHMY, A. R. & PIERREPOINT, C. G. (1970) Testosterone Metabolism in the Dog Prostate with Regard to its Growth and Function. In *Some Aspects of the Aetiology and Biochemistry of Prostatic Cancer.* Ed. K. Griffiths and C. G. Pierrepoint. Cardiff: Alpha Omega Alpha Publishing. p. 88.

HAWKINS, R. A., FREEDMAN, B., MARSHALL, A. & KILLEN, E. (1975) Oestradiol-17β and Prolactin Levels in Rat Peripheral Plasma. *Br. J. Cancer*, **32**, 179.

HUGGINS, C. (1963) The Hormone-dependent Cancers. *J. Am. med. Ass.*, **186**, 481.

HUGGINS, C., BRIZIARELLI, G. & SUTTON, H. (1959) Rapid Induction of Mammary Carcinoma in the Rat and the Influence of Hormones on the Tumours. *J. exp. Med.*, **109**, 25.

HUGGINS, C. & MAINZER, K. (1957) Hormonal Influence on Mammary Tumours of the Rat. II. Retardation of Growth of a Transplanted Fibroadenoma in Intact Female Rats by Steroids in the Androstane Series. *J. exp. Med.*, **105**, 485.

JENKINS, J. S. & MCCAFFERTY, V. M. (1974) Effect of Oestradiol-17β and Progesterone on the Metabolism of Testosterone by Human Prostate Tissue. *J. Endocr.*, **63**, 517.

KING, R. J. B., GORDON, J. & HELFENSTEIN, J. E. (1964) The Metabolism of Testosterone by Tissues from Normal and Neoplastic Rat Breast. *J. Endocr.*, **29**, 103.

KITAY, J. I., COYNE, M. B. & SWYGERT, N. H. (1970) Influence of Gonadectomy and Replacement with Oestradiol or Testosterone on Formation of 5α-reduced Metabolites of Corticosterone by the Adrenal Gland of the Rat. *Endocrinology*, **87**, 1257.

MILLER, W. R., FORREST, A. P. M. & HAMILTON, T. (1974) Steroid Metabolism by Human Breast and Rat Mammary Carcinomata. *Steroids*, **23**, 379.

SALIH, H., FLAX, H. & HOBBS, J. R. (1972) *In vitro* Oestrogen Sensitivity of Breast Cancer Tissue as a Possible Screening Method for Hormonal Treatment. *Lancet*, i, 1198.

SCHRIEFERS, H. (1967) Factors Regulating the Metabolism of Steroids. *Vitams Horm.*, **25**, 271.